# Experimental Research on Data Synchronous Acquisition Method of Subsidence Monitoring in Submarine Gas Hydrate Mining Area

**DOI:** 10.3390/s19194319

**Published:** 2019-10-06

**Authors:** Jiawang Chen, Chen Cao, Yongqiang Ge, Huangchao Zhu, Chunying Xu, Yan Sheng, Lieyu Tian, Hanquan Zhang

**Affiliations:** 1 Ocean College, Zhejiang University, Zhoushan 316021, China; arwang@zju.edu.cn (J.C.); cc666@zju.edu.cn (C.C.); Ge_yongqiang@zju.edu.cn (Y.G.); 21634119@zju.edu.cn (H.Z.); 2 Guangzhou Marine Geological Survey, Guangzhou 510075, China; shengyan@hydz.cn (Y.S.); tianlieyu23@163.com (L.T.); 13660367888@139.com (H.Z.)

**Keywords:** data acquisition, time synchronization, subsidence monitoring, submarine gas hydrate mining area

## Abstract

The data synchronous acquisition is crucial to the seafloor subsidence monitoring system for gas hydrate mining areas based on microelectromechanical sensors (MEMS). Because the independent and high-precision time reference sources on land cannot be used on the seafloor, especially in the deep sea, a relative time synchronization method based on input/output (I/O) and controller area network (CAN) bus was proposed to realize the internal time synchronization of the system. To demonstrate the feasibility of the proposed method, tests including the deformation test of the MEMS sensor array under high pressure, synchronous accuracy test, and landslide and collapse simulation tests were carried out. The synchronization method was performed once every 24 h, and the time drift was reduced to 0.38 ms from more than 30 ms, demonstrating that method can achieve consistent internal time of the system. The method does not require additional hardware devices and has adjustable accuracy.

## 1. Introduction

Natural gas hydrate is an important green (low carbon) energy source [1], of which the solid phase only exists in low temperature and high pressure [2]. It is easy to burn and is commonly called “combustible ice” [2,3,4,5]. Submarine gas hydrates bring new energy prospects to humans, while it also presents a hazard for the environment and human beings. If the conditions change, the hydrate releases methane gas and the physical properties of the seafloor sediment are also altered. As a result, the mechanical properties of seabed sediments are greatly reduced and the seabed is softened, causing large-scale submarine landslides, collapses, and the destruction of subsea engineering facilities [6,7,8]. Therefore, in-situ and long-term monitoring of subsidence during the exploitation of submarine gas hydrates is of great significance for the study of the formation mechanism of submarine landslides, hydrate environmental impact assessment, and early warning.

At present, the means of seafloor subsidence monitoring are mainly through satellite remote sensing and single-beam and multi-beam devices, among which acoustic measurements, such as multi-beam, are the most common method. These acoustic devices need to be mounted on a measuring platform, such as a surveying ship, a remotely controlled vehicle (ROV) or an autonomous underwater vehicle (AUV). These monitoring methods cannot achieve in-situ, long-term monitoring due to high costs [9,10,11,12].

In Japan, pressure sensors and three-component servo-accelerometers were used for subsidence monitoring, but only for a single-point measurement to monitor the settlement of a certain point [8,13,14] In recent decades, the technology of microelectromechanical systems (MEMS) has developed rapidly. MEMS inclinometers and accelerometers have been widely used for land subsidence and landslide detection on land. The Measurand Company of Canada produces a MEMS accelerometer array called the Shape Accel Array (SAA). The SAA measures the shapes of the path in the borehole and structure and monitors the deformation of the land-based structure and slope [8,15]. However, there are few articles on submarine in-situ surface mapping and multiple-point subsidence monitoring at home and aboard.

Besides, there are many researches on data acquisition in multi-node and distributed systems, as well as time synchronization. Most of these systems are in the form of a single bus, and few articles use the combination of inter-integrated circuit (IIC) bus (short distance, high speed) and controller area network (CAN) bus (long distance, multi-node) to achieve high-speed and long-distance data transmission [11,16,17]. Meanwhile, underwater sensor networks have attracted more and more attention, and are widely used in marine environmental monitoring, resource exploration, target detection, tracking, and positioning [18,19].

It is meaningful to match the data collected by the distributed sensor nodes with the time information. Data with same time collected is also the basis for implementing technologies such as network cooperative work. Generally, time synchronization is mainly based on the use of independent and high-precision clock reference sources, such as a global positioning system (GPS) or Beidou time-scaler, which are commonly used on land. The method makes it easy to achieve time synchronization of the system, where the requirements of the clock source are very demanding. However, the seawater shields electromagnetic waves, light waves, and other signal transmissions. Therefore, many underwater time synchronizations must obtain a synchronization signal by setting a receiving base station on the sea level through an optical fiber or a cable. Li et al. used the satellite signal of the GPS/Beidou dual reference source received by the precision time protocol (PTP) master time (base station) as the time source. The decoded output network time protocol (NTP) synchronization signal and PTP synchronization signal were transmitted through the fiber optic cable to obtain an accurate time [20,21]. Moreover, there is also a time synchronization technology based on IRIG-B code, which is a time standard code developed to realize information exchange between the fields. The IRIG-B code combines the advantages of pulse timing and serial message timing, of which encoding has high synchronization precision and can reach microsecond level. However, the generation and reception of IRIG-B code is very complicated, requiring special equipment and high cost [22,23]. In addition, few researches have been done on time synchronization in the deep sea where clock sources cannot be used.

In this article, the topography of 30 m × 30 m around the natural gas hydrate mining area was taken as the research object. The multi-node data acquisition technology and system time synchronization method were taken as the main research contents. The remainder of the article is organized as follows. Section 2 describes the design of subsidence monitoring system based on MEMS sensor. Section 3 gives a description of two-stage data acquisition system based on IIC bus and CAN bus. Section 4 presents the design of relative time synchronization method for data synchronous acquisition. Section 5 presents a series of tests including synchronous testing and error evaluation, submarine subsidence simulation, and sensor array pressure test, which were carried out to demonstrate the performance of the data synchronous acquisition method. At last, Section 6 presents the conclusions and further research.

## 2. Design of Subsidence Monitoring System Based on MEMS Sensor for Gas Hydrate Mining Area

In-situ and long-term monitoring are two major difficulties of the system. In this article, multiple observation points were set, each of which can perform high-accuracy measurements, and network communication is required among observation points.

**Figure 1:**
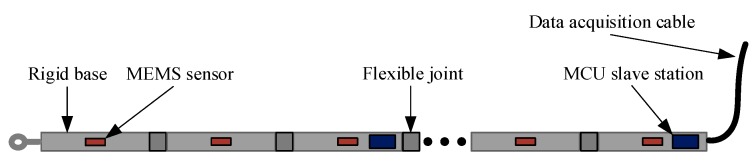
Microelectromechanical sensor (MEMS) array structure.

The displacement measurement is an effective method to measure the subsidence of seafloor topography. In this article, the MEMS attitude sensor (JY901 Weite Intelligent Co., Ltd. Shenzhen, China) was used to collect the acceleration information of each node, and the displacement of each point was obtained by filtering and integration algorithm to construct a three-dimensional seafloor subsidence model. The shape between the sensors is fitted with a short straight line, and all the sensors on the array need to maintain a non-twisted state during operation. If the array can twist freely, the directions between subsidence and uplift cannot be distinguished when the sensor is reversed. Therefore, the sensors were arranged in a strip-shaped rigid base (polyamide) with segments, where the joints were connected by flexible joints (composite rubber). MEMS sensors, microcontroller unit (MCU) (STM32F103ZE) slave stations, and the data acquisition cable are on the array. The structure of MEMS sensor array is shown in Figure 1.

**Figure 2:**
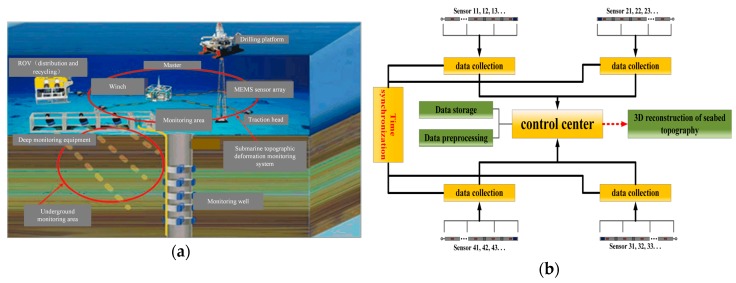
(**a**) MEMS sensor array submarine layout; (**b**) Data synchronous acquisition system diagram.

Four MEMS sensor arrays were placed in the shape of ‘X’ in the gas hydrate mining area, as shown in Figure 2a. There were 21 MEMS attitude sensors on each array. The controller was set in the center of the entire system and was responsible for the communication, storage, and power supply of the whole system. The collected data was finally sent to the controller to reconstruct the three-dimensional model of the seafloor subsidence. The data synchronization acquisition system diagram is shown in Figure 2b.

## 3. Two-Stage Data Acquisition System Based on IIC (Inter-Integrated Circuit) Bus and CAN (Controller Area Network) Bus

The data collection in this system has two main challenges. First, the number of sensor nodes is large. If each node is independently connected to the controller, the stability and reliability of the system would be reduced. Second, long-distance data collection needs to ensure real-time data collection.

**Figure 3:**
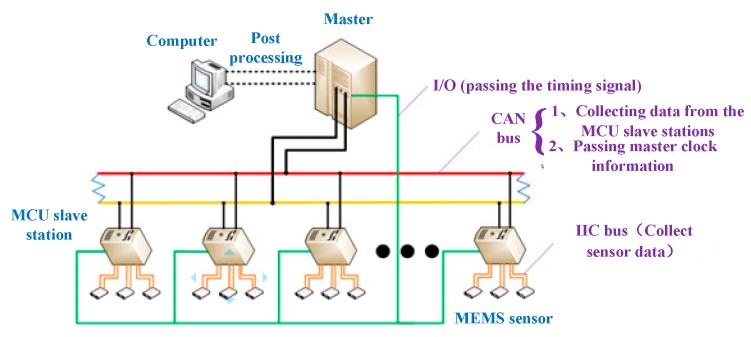
Two-stage data acquisition and transmission system based on the inter-integrated circuit (IIC) bus and controller area network (CAN) bus.

In order to solve the difficulty of data collection, the set one MCU slave station for every three sensors. The data synchronous acquisition and transmission system adopted the master-slave structure, the master station (controller) was responsible for collecting, storing, and pre-processing, while the slave was responsible for the acquisition of the acceleration sensor data. The data transmission between the sensors and the slave used the IIC bus (the first-stage data acquisition and transmission system). The data of the slaves and the masters were transmitted through the CAN bus (the second-stage data acquisition and transmission system). Two-stage data acquisition and transmission system are shown in Figure 3.

### 3.1. The First-Stage Data Acquisition and Transmission System Based on IIC Bus

**Figure 4:**
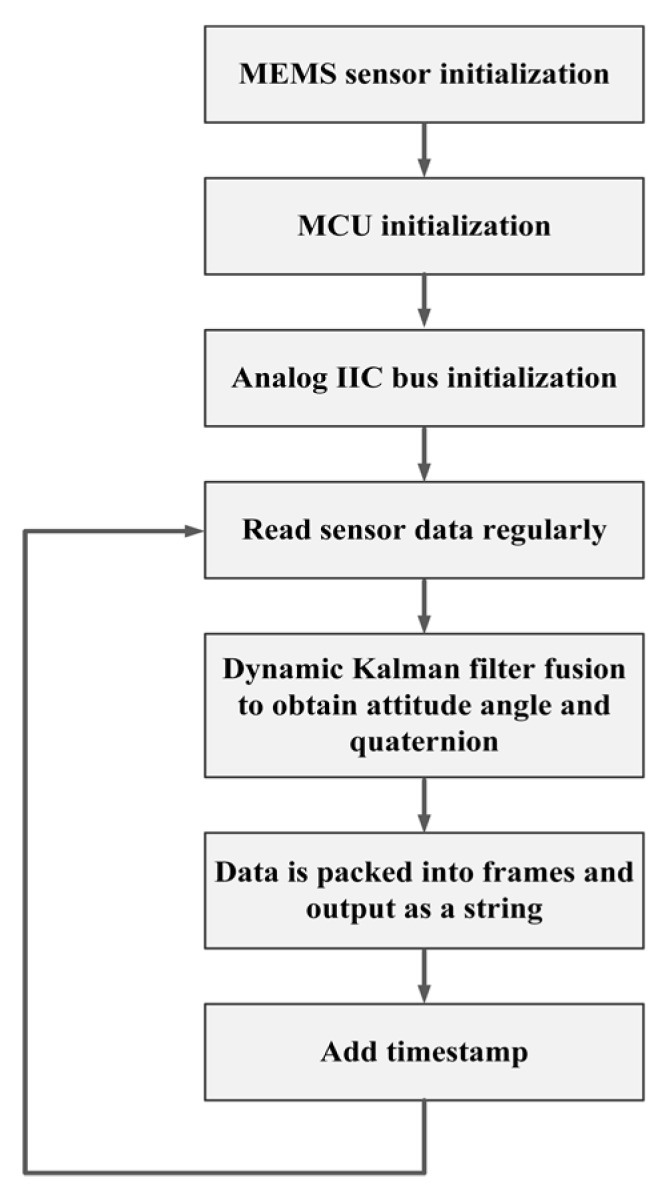
Data processing program flowchart.

The slaves need to control the time of data collection, frequency, and transmission sequence of the sensor node. After collecting the data of the corresponding node, timestamp and identification characters were added at the end of the data. “#” was used as the start character and “$” was used as the terminator. The data was preprocessed, including filter and data frame packing. It was converted as a string, facilitating the extraction and processing of the data in the three-dimensional dynamic reconstruction of the subsidence. The data processing program of the slave is as shown in the Figure 4.

### 3.2. Figures, Tables, and Schemes

**Figure 5:**
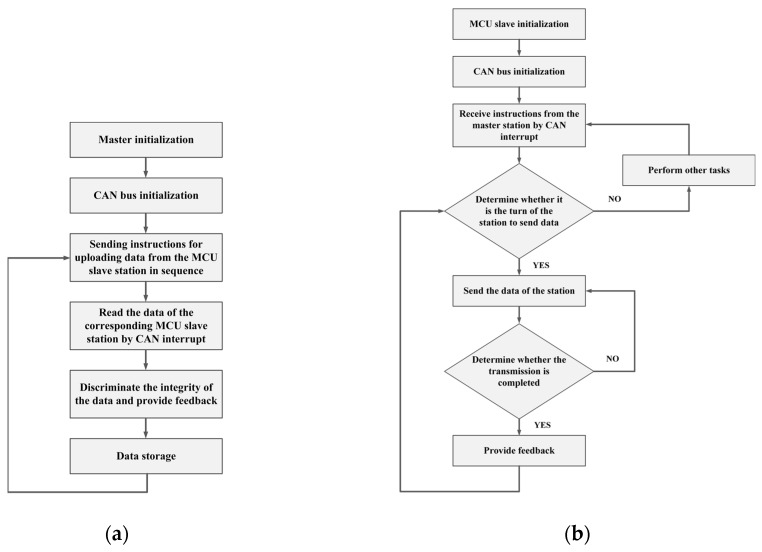
(**a**) Master station flow diagram; (**b**) Slave station flow diagram.

The MCU controller adopts the form of master-slave stations. At first, the master sends a verification code to the slaves via the CAN bus, which is used to designate a slave to send data to the master. Once the slave sends the data to the CAN bus, the master will receive the data via the CAN interrupt and determine the integrity of the data. If the data is complete, it will be stored for processing and analyzing. In addition, the slave notifies the master at the end of transmission, and the master also informs the slaves when the data is received. The closed-loop feedback can be formed to ensure efficient and orderly transmission. The flow diagrams of the master and the slave are as shown in the Figure 5a,b.

## 4. Design of Relative Time Synchronization System

The reconstruction of the three-dimensional subsidence dynamic model requires high simultaneity of data. If the time of the data collected by each sensor is inconsistent, the reconstructed three-dimensional subsidence dynamics would be inconsistent with the actual seabed as well. Therefore, time synchronization of all the accelerometer data on the four arrays is required, which means time synchronization of all the slaves needs to achieve a better synchronization accuracy. The time of the electronic component is generally provided by a quartz crystal oscillator. The quartz crystal oscillator generates a fundamental frequency, which is converted into a local time signal of the node by frequency division and frequency multiplication. However, there are slight differences in the production of crystal oscillators [24]. Meanwhile, environmental conditions such as temperature can also cause changes in the oscillation frequency of the crystal oscillator [25]. Realizing the synchronous acquisition of the data of subsidence in the seabed environment without adding any extra hardware devices is very meaningful.

### 4.1. The First-Stage Data Acquisition and Transmission System Based on IIC Bus

**Figure 6:**
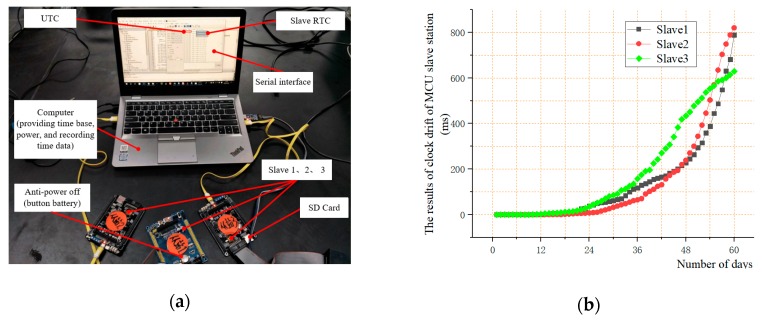
(**a**) The test of clock drift of slave station; (**b**) The results of clock drift of slave station.

STM32F103ZET6 was selected as the MCU of the slave and the time drift of the slave was tested by comparing the real-time clock (RTC) of the MCU with the coordinated universal time (UTC). The MCU continuously runs for 60 days without interruption and automatically saves RTC information and UTC information at 9:00 every day. As Figure 6a shows, three slaves were tested. The results of the test are as shown in the Figure 6b. Within the first 20 days, the time drift of the slave was small, with a maximum of 14.3 ms. However, the drift increased rapidly over time and 60 days later, the drift reached 820.28 ms. Moreover, the drift rates of the three slaves are inconsistent.

### 4.2. The Second-Stage Data Acquisition and Transmission System Based on CAN Bus

**Figure 7:**
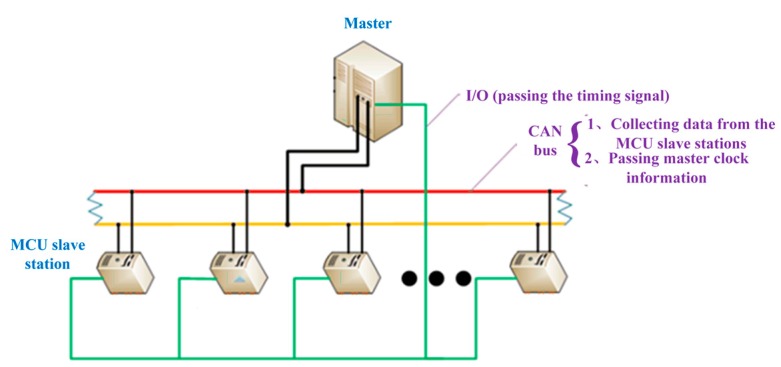
System relative time synchronization schematic.

Considering the particularity of the system submarine working environment, it is unable to communicate with the maritime base station under the deep sea nor is it suitable to add more hardware devices to achieve time synchronization. A relative time synchronization method was designed to ensure the relative synchronization of the slaves and master in the system. When the slaves collect the sensor data, a timestamp is added to each group of data. It is beneficial to implement time synchronization and data extraction in the subsequent three-dimensional reconstruction. The time source for synchronization of each slave is periodically synchronized with the time of the master. The CAN bus is a serial bus, which means the information on the CAN bus is transmitted sequentially in order. In other words, the information cannot be transmitted to each sensor node at the same time. On the other hand, the signal on the input/output (I/O) can be synchronously transmitted [26]. Therefore, the relative synchronization was performed as follows: The master clock information was transmitted through the CAN bus in advance, and the timing signal was simultaneously sent from the I/O to the slaves. The system relative time synchronization schematic is shown in Figure 7.

**Figure 8:**
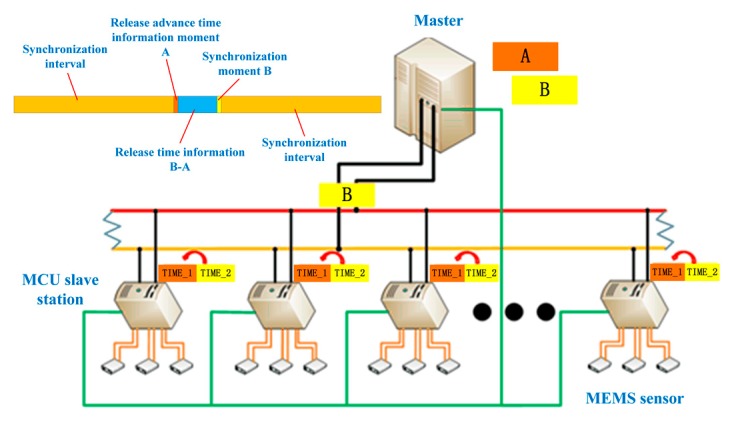
System relative time synchronization principle diagram.

When the master reaches a certain moment A, the moment B (B is later than A) will be transmitted to each slave through the CAN bus in advance and stored as a variable. The interval between B–A is to ensure that the master clock information can be passed to each slave during this time. For example, TIME_2. TIME_1 is a variable of the local time of the slave. Then, when the time of the master reaches the moment B, a trigger signal is sent to each slave through the I/O. After that, the variable TIME_1 is covered by the variable TIME_2. The schematic is shown in the Figure 8.

The system relative time synchronization method involves using each slave to periodically synchronize with the master. The master sequentially sends the time information through the CAN bus in advance, then sends the trigger signal through the I/O synchronization. In this way, each slave is synchronized with the clock source of the master, so that the system can ensure the relative synchronization of the internal time.

**Figure 9:**
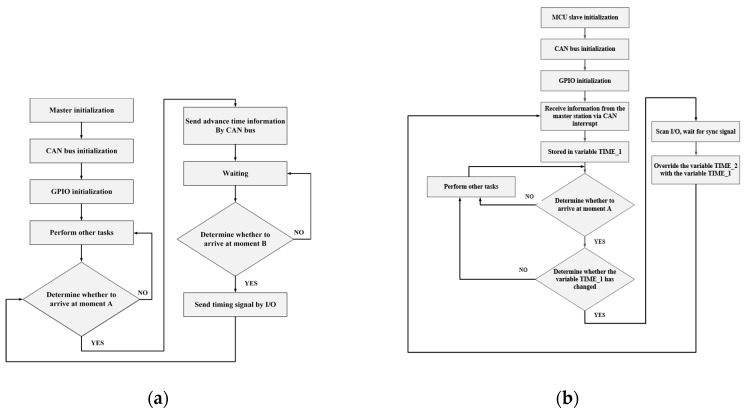
(**a**) Time synchronization master flow diagram; (**b**) Time synchronization slaver flow diagram.

After the initialization is completed, the master will send the advance time to the slaves through the CAN bus when time reaches to a moment set. When the time of the master reaches the advanced time of the transmission, the master will send the synchronous trigger signal through I/O. Then, the master enters the next wait and loops. The slave receives the advance time sent by the master through the CAN interruption. Identically, it is received in order and stored in a certain variable. The slave will periodically check this variable to determine whether the value of the variable has changed. If there is no change, it continues the task of sending or receiving data. Otherwise, it will stop the data receiving and sending, scanning the I/O continuously until the arrival of trigger signal is received. Once this trigger signal is received, the defined variable is overwritten. Time synchronization master and slaver flow diagrams are shown in Figure 9a,b.

## 5. Tests and Results

The test of the data synchronous acquisition system was divided into three parts, namely the pressure test, synchronous accuracy test, and landslide and collapse simulation test. The pressure test simulates the deep-sea environment and tests the system’s ability to work under high pressure. The synchronization accuracy test was performed to verify the effect of synchronization, to test the synchronization error, and to analyze the cause of the time deviation. The landslide and collapse simulation test was carried out to verify the feasibility and effectiveness of data synchronous acquisition system. The shapes of seabed terrain can be monitored with real-time.

### 5.1. Deformation Test of MEMS Sensor Array Under High Pressure

**Figure 10:**
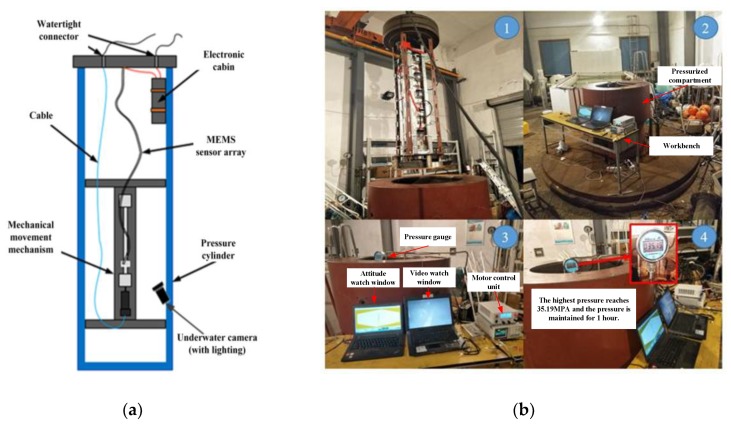
(**a**) The device schematic of the high-pressure test; (**b**) The picture of the high-pressure test.

The data synchronous acquisition system works on the seabed. The main difficulties are sealing and withstanding high pressure. The solution for the pressure test is to place the sensor array (including the electronics compartment) into a high-pressure cylinder (as shown in Figure 10a,b). One end of the array is fixed to the high-pressure cylinder cover and the other end is fixed to the screw slider. The mechanical motion device is used to generate the shapes of the sensor array. The mechanical motion device is composed of an underwater pressure-resistant motor and a screw nut mechanism. The rotation speed and steering of the motor are driven by a controller. The underwater camera was used to capture the shape changes of the array, with a ruler placed next to the sensor array. The shapes of sensor array were reconstructed from the acceleration data. The results in the air and under high pressure were compared, which demonstrates the feasibility and accuracy of the application of the distributed data synchronous acquisition system in the seabed.

**Figure 11:**
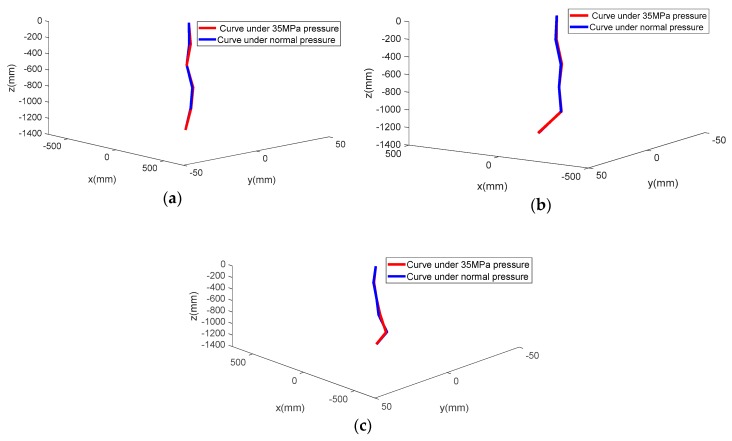
(**a**), (**b**), and (**c**) are the curves obtained from the two states. Red lines are the measurement curves from the MEMS accelerometers under 35 MPa pressure and blue lines are the measurement curves from the MEMS accelerometers under normal pressure.

Table 1 is the measurement error of the three shapes between 35 MPa pressure and normal pressure. From Figure 11 and Table 1, strong agreement is demonstrated between the measured data from the MEMS accelerometers under 35 MPa pressure and normal pressure. In the three shape tests, the maximum RMSE was 2.26 mm, and maximum absolute error was 5.33 mm. It can be inferred that this data synchronous acquisition system can be used in a deep sea environment.

### 5.2. Synchronous Accuracy Test

**Figure 12:**
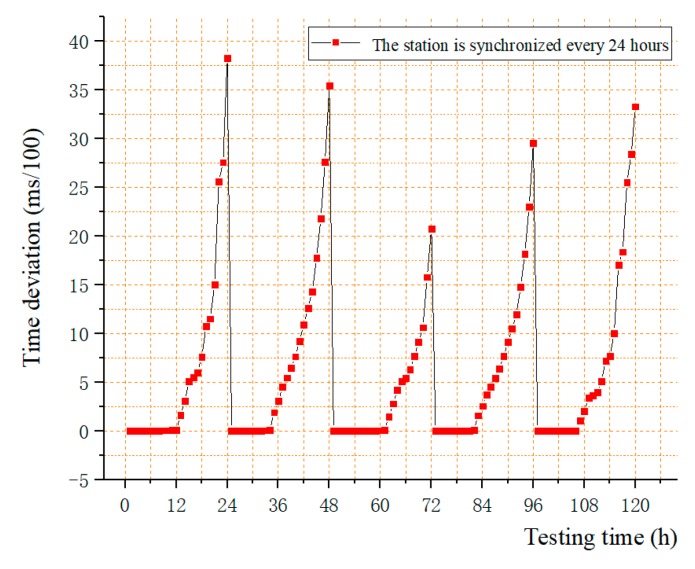
The results of synchronous accuracy test (the slave is synchronized every 24 h).

The longer the data acquisition system runs, the worse the synchronization will be. The time synchronization method of the data acquisition system was carried out for slave station every 24 h, and the results are as shown in Figure 12. In the first 12 h, the amount of time drift was smaller and closer to 0, but it does exist. However, from 12 to 24 h, the amount of time drift greatly increased because it is an error accumulation. It is conceivable that without synchronization, time drift will continue to increase. Therefore, in the case of synchronization, the average time drift was 3.16 ms in 24 h.

### 5.3. Synchronization Error Analysis

**Figure 13:**
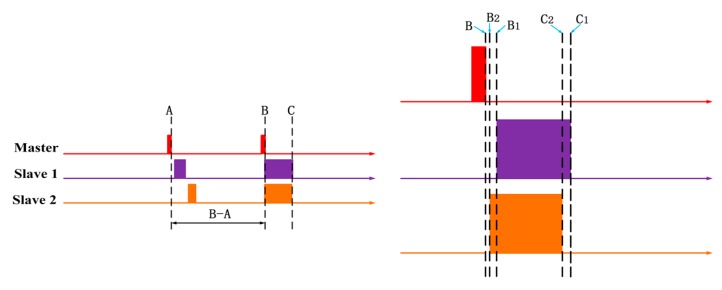
The errors of time synchronization method.

The errors that may occur in the time synchronization of the data acquisition system were evaluated using three main sources: (1) The deviation of the clock from each slave in the synchronization interval, (2) the deviation of the slave executes the synchronization code, and (3) the deviation of the response time of each slave from the master trigger signal. As shown in the Figure 13, A is the moment when the master sends the advance time information, and B is the moment when the master sends a trigger signal through I/O. B–A (from moment A to moment B) is to ensure that the advance time information can be communicated to each slave ensuring no error in time synchronization. C is the moment when the synchronization is completed. Errors mainly occur between moment B and moment C or after moment C.

#### 5.3.1. Test of the Time (Ci-Bi) Required to Execute the Synchronization Code

**Figure 14:**
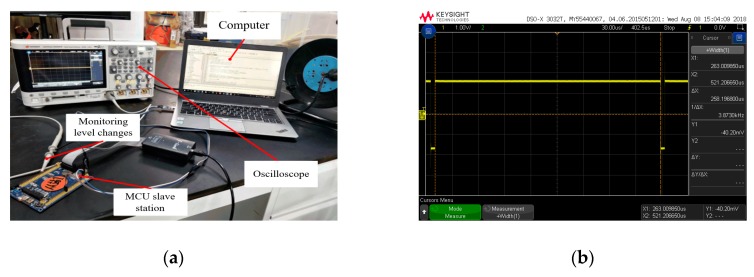
(**a**) The test for the time of synchronous code; (**b**) One of the results.

The time required for different controllers to execute the same program may be different, and the time required for the same controller to execute the same program at different times is also different. In this test, three STM32 MCUs executed the same sync code by setting a high-level signal before executing the synchronization code, setting a low-level signal at the end, and then using the oscilloscope to detect the duration of high-level signal to obtain the time required to execute the code, as shown in Figure 14. Every MCU randomly detected 30 times.

**Figure 15:**
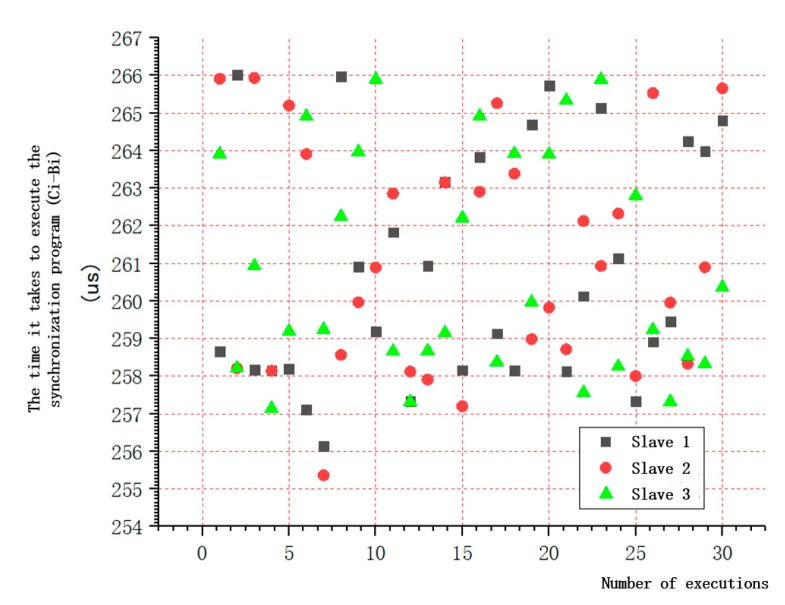
The results of the time required for single slave to execute synchronous code.

The results are illustrated in Figure 15. The longest time was 266.376 us, and the shortest time was 258.0164 us. The errors of the time (Ci-Bi) required to execute the synchronization code was less than 8.3596 us (the longest time minus the shortest time), which indicates that the time taken by different MCU slaves or the same MCU slave to execute the same synchronization code was deviated. The deviation was caused by various factors including the length of the code, the hardware of the MCU itself, the operating status and environment, etc.

#### 5.3.2. Test of the Time Deviation (Bi-Bj) of the Response between the Two Slaves to the Trigger Signal of the Master

**Figure 16:**
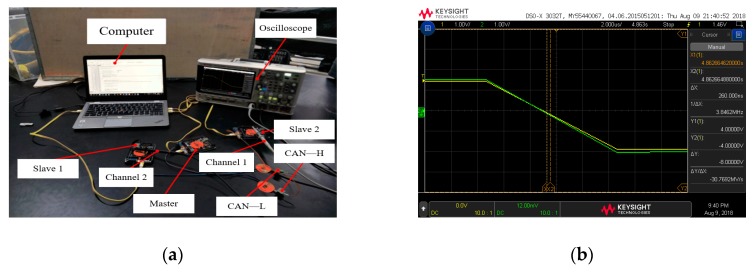
(**a**) The test for time deviation of the response between the two slaves to the trigger signal of the master; (**b**) One of the results.

**Figure 17:**
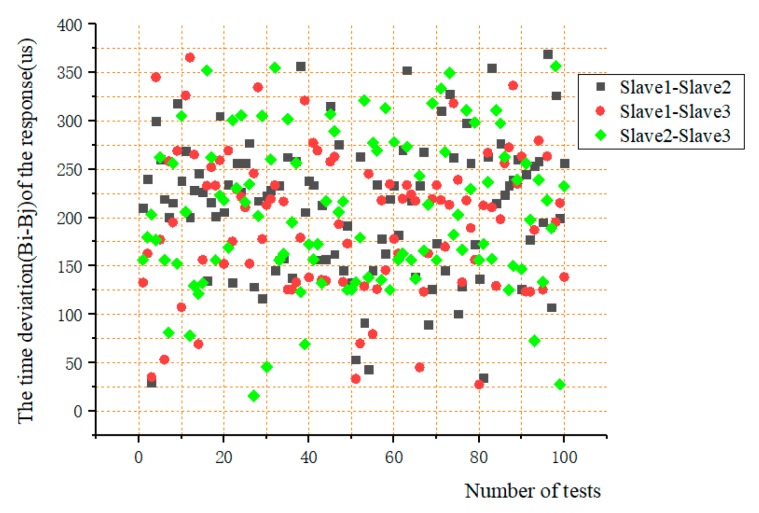
The results of the time differences required for two slaves to execute synchronous code.

When the master sends synchronization trigger signal to each slave through I/O, each slave determines whether to perform time synchronization by executing a piece of synchronous code. The response time of each slave to the trigger signal of the master is not the same. In this test, a high-level trigger signal was sent every 10 min through the master, and the advance time information was transmitted through the CAN bus. Three slave pairs (Slave1 and Slave2, Slave1 and Slave3, Slave2 and Slave3) received the advanced time information, then scanned the I/O to react to the trigger signal. Similarly, the high-level signal was set before the slave executes the synchronization code, and the level changes of the two slaves were respectively detected by the two channels of the oscilloscope. In the meantime, the deviation (B1-B2) was measured. Each two slaves were tested 100 times. Figure 16 and Figure 17 show the time deviation of the response of between the two slaves to the trigger signal of the master.

From the Figure 17, it can be seen that the deviation values were less than 400 us, mainly ranging from 100 us to 300 us. It is concluded that the time for each slave to respond to the main trigger signal was basically the same, of which the deviation was very small.

#### 5.3.3. Synchronization Error Evaluation

The three error sources of the data acquisition system were tested respectively and the distribution of the error range was obtained, as shown in Table 2.

The drift of the clock was tested according to the 24 h interval. It can be seen that the error of the data acquisition system mainly came from the drift of the clock from slave in the interval of synchronization, reaching 3.16 ms in 24 h. The effects of the second and third items were small, less than 8.5 us and 375 us, respectively. However, as the synchronization frequency increases, the impact of these two parts would increase, which is related to the requirements for synchronization accuracy.

The synchronization accuracy increases with the shortening the interval. The accuracy is also affected by the environment such as temperature and vibration. This test was measured in a relatively stable indoor environment, so the actual drift value was larger.

### 5.4. Synchronization Error Analysis

**Figure 18:**
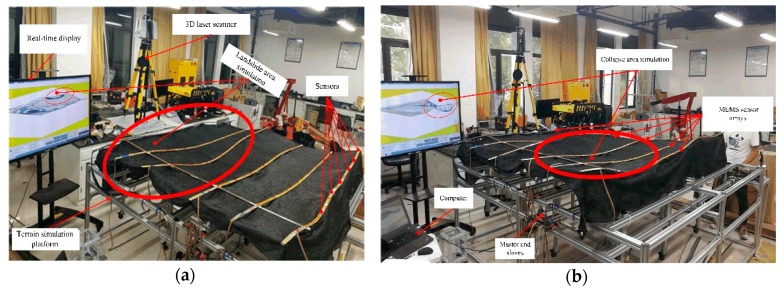
(**a**) Landslide phenomenon simulation test; (**b**) Collapse phenomenon simulation test.

**Figure 19:**
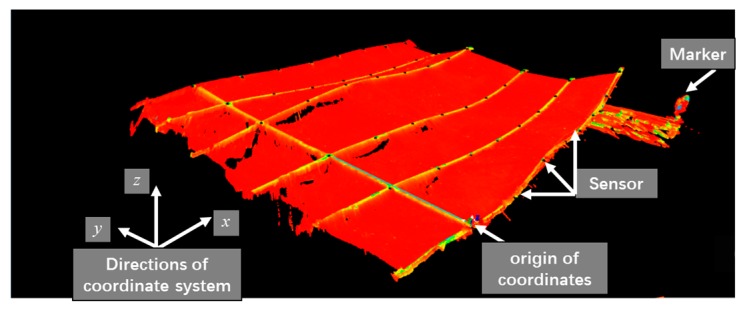
Point cloud data obtained by 3D laser scanner.

The submarine landslide and collapse phenomenon are the key monitoring contents of the MEMS sensor array system, which is characterized by rapid occurrence and large amount of topographic settlement. In response to this feature, a terrain simulation platform in laboratory was set up which could quickly deform and test the system’s ability to monitor extreme geological phenomena (collapse, landslide, etc.) on the seabed. By programming the motor control slide screw matrix (16), the maximum speed was used to simulate the landslide and collapse. The upper computer displayed the deformation process in real-time and checked the synchronization of data acquisition, as shown in Figure 18. The three-dimensional laser scanner obtained a large amount of point cloud data. The coordinates of the sensor points were obtained from the point cloud data which was processed by the commercial software called Cyclone. Figure 19 shows the point cloud data obtained by three-dimensional (3D) laser scanner in the Cyclone.

#### Results Analysis and Conclusions

The coordinates of the position of the sensor calculated by the data collected by the sensor in MATLAB were compared with the coordinate information of the position of the sensor processed in the cyclone software (considered to be the true shape). The reconstructed shape was basically consistent with the real shape. The error analysis is shown in Table 3.

From the figure and data analysis, the maximum mean square error of the two phenomena is 1.02 cm while the maximum error is 1.32 cm, which means that the accuracy of 3D reconstruction is high. It is obvious that the submarine terrain deformation monitoring system based on MEMS sensing array has high monitoring accuracy for the deformation process, with high deformation speed and large deformation. In addition, the measurement accuracy also shows that the data collected by each sensor node has good time synchronization. The host computer exhibited a certain lag which was about 0.2s~0.8s, and part of this time was due to the processing of data by the MATLAB software.

## 6. Summary and Outlook

The article proposed a high-speed synchronous acquisition method for multi-node, distributed systems, which has the following characteristics:

(1) The data acquisition of MEMS sensor was carried out by combining the two stages of IIC bus and CAN bus, where pre-processing, such as feedback control and time stamping, can effectively realize data collection and transmission, which greatly facilitates post-processing of data.

(2) According to the particularity of the submarine working environment, a system-time synchronization scheme was proposed, which can realize the time synchronization between different sensor nodes. This method can also be applied to the case where it is impossible to use a universal independent high-precision time reference source, such as the GPS/Beidou time scale.

(3) Additional hardware devices are not necessary for achieving synchronous acquisition, which improves the stability of the system.

(4) The accuracy of the synchronization can be adjusted by the synchronization interval.

Compared to the methods of acoustic measurement, such as multi-beam method, the data synchronization acquisition system and monitoring method have advantages of strong anti-interference and anti-deformation ability, independent measurement, etc. In addition, the system has good expandability and can be used for monitoring of various deformed surfaces such as engineering and medicine in the future. The system will be tested in the South China Sea gas hydrate mining area next year and we will continue to report it.

## Figures and Tables

**Table 1:** The measurement errors.

	Mean Absolute Error (mm)	Root Mean Square Error (mm)	Maximum Absolute Error (mm)
Shape1	4.62	5.33	6.72
Shape2	1.53	2.26	2.18
Shape3	2.06	4.32	4.66

**Table 2:** The results of synchronous accuracy test (the station is synchronized every 24 h).

Source of Error	Range of Error
The drift of the clock from slave in the interval of synchronization	3.16 ms
The deviation of the slave executes the synchronization code	<8.5 us
The deviation of the response time of slave from the master I/O trigger signal	<375 us

**Table 3:** Analysis of simulation measurement errors of landslide and collapse.

	Mean Square Error (cm)	Maximum Error (cm)	Maximum Deformation (cm)	Maximum Tilt Angle (°)	Minimum Tilt Angle (°)
Landslide	0.85	1.26	18.3	28.03	1.25
Collapse	1.02	1.32	23.7	20.95	1.62
